# Supplementary material for: Kinetochore mutations and histone phosphorylation pattern changes accompany holo- and macro-monocentromere evolution
Source: Nat Commun. 2025 Dec 20;16:11332. doi: 10.1038/s41467-025-67524-8 (PMC12722358; doi:10.1038/s41467-025-67524-8)
Supplement: Supplementary file 1 — Supplementary Information [file 41467_2025_67524_MOESM1_ESM.pdf]

Supplemental Information

Kinetochore mutations and histone phosphorylation pattern changes accompany holo- and macro-monocentromere evolution

Yi-Tzu Kuo, Pavel Neumann, Jianyong Chen, Jörg Fuchs, Veit Schubert, Katrin Kumke, Mariela Analia Sader, Michael Melzer, Zihao Zhu, Axel Himmelbach, Heiko Hentrich, Jiří Macas, Andreas Houben

Supplementary Table 1 Genome assembly of *Cha. luteum*

Statistics of genome assembly	Primary assembly	Assembly after purge_dups
# contigs	4,087	649
Largest contig	19,839,389	19,839,389
Total length	1,556,940,134	744,551,200
N50	1,631,558	2,111,717
N75	663,828	1,228,881
L50	192	87
L75	564	205
GC (%)	40.51	41.13
Complete BUSCOs	3094 (95.6%)	3001 (92.7%)
Complete and single-copy BUSCOs	895 (27.7%)	2657 (82.1%)
Complete and duplicated BUSCOs	2199 (68.0%)	344 (10.6%)
Fragmented BUSCO	106 (3.3%)	126 (3.9%)
Missing BUSCO	36 (1.1%)	109 (3.4%)
Total BUSCO groups searched	3,236	3,236

* The BUSCO dataset: liliopsida_odb10

Supplementary Table 2 Hi-C scaffolding and CENH3-ChIPseq determined centromere position and size on the top 22 scaffolds of *Cha. luteum*

Scaffold	Scaffold size (bp)	Start of centromere	End of centromere	Centromere size (bp)
Clu_scaffold_1	68,265,395	27,141,008	42,438,856	15,297,848
Clu_scaffold_2	65,513,375	-	-	-
Clu_scaffold_3	60,524,292	14,379,053	25,678,443	11,299,390
Clu_scaffold_4	60,505,644	23,695,211	35,424,698	11,729,487
Clu_scaffold_5	56,158,475	15,525,124	26,162,203	10,637,079
Clu_scaffold_6	47,624,097	-	-	-
Clu_scaffold_7	43,992,422	-	-	-
Clu_scaffold_8	40,609,926	-	-	-
Clu_scaffold_9	39,688,115	-	-	-
Clu_scaffold_10	31,882,672	964,644	13,258,378	12,293,734
Clu_scaffold_11	31,274,261	-	-	-
Clu_scaffold_12	24,971,802	356,741	9,961,965	9,605,224
Clu_scaffold_13	24,787,866	-	-	-
Clu_scaffold_14	22,080,910	-	-	-
Clu_scaffold_15	16,898,067	-	-	-
Clu_scaffold_16	14,908,028	-	-	-
Clu_scaffold_17	14,449,349	2,024,067	13,887,568	11,863,501
Clu_scaffold_18	13,038,212	-	-	-
Clu_scaffold_19	12,504,476	-	-	-
Clu_scaffold_20	11,731,529	661,293	10,240,432	9,579,139
Clu_scaffold_21	11,184,362	-	-	-
Clu_scaffold_22	10,419,296	-	-	-

Supplementary Table 3 Repetitive composition of the *Cha. luteum* genome analyzed using RepeatExplorer

Repeat	Genome proportion (%)	
Mobile element_Class I_LTR_Ty1 Copia_Ale		2.36
Mobile element_Class I_LTR_Ty1 Copia_Angela		5.00
Mobile element_Class I_LTR_Ty1 Copia_Bianca		0.79
Mobile element_Class I_LTR_Ty1 Copia_Ikeros		0.18
Mobile element_Class I_LTR_Ty1 Copia_TAR		0.20
Mobile element_Class I_LTR_Ty1 Copia_Tork		0.37
Total Ty1 Copia	8.90	
Mobile element_Class I_LTR_Ty3 Gypsy_non-chromovirus_Athila		3.77
Mobile element_Class I_LTR_Ty3 Gypsy_non-chromovirus_Ogre		0.85
Mobile element_Class I_LTR_Ty3 Gypsy_non-chromovirus_Retand		5.05
Mobile element_Class I_LTR_Ty3 Gypsy_chromovirus_CRM		1.20
Mobile element_Class I_LTR_Ty3 Gypsy_chromovirus_Tekay		1.32
Mobile element_Class I_LTR_Ty3 Gypsy_chromovirus_Tcn1		0.01
Total Ty3 Gypsy	12.20	
Mobile element_Class I_LTR_unclassified		0.77
Mobile element_Class I_LINE		0.80
Mobile element_Class I_Other Class I		0.06
Mobile element_Class II_DNA transposone_TIR_EnSpm CACTA		0.28
Mobile element_Class II_DNA transposone_TIR_hAT		1.51
Mobile element_Class II_DNA transposone_TIR_Ivana		0.09
Mobile element_Class II_DNA transposone_TIR_MuDR Mutator		0.08
Mobile element_Class II_DNA transposone_TIR_PIF Harbinger		0.22
Total DNA transposon	2.18	
Tandem repeat_Satellite DNA		12.12
Tandem repeat_rDNA		1.58
Unclassified		10.13
Total repeat	48.74	

Supplementary Table 4 Percentage of CENH3 signal colocalizing with *Chama* repeats measured in 16 *Cha. luteum* root interphase nuclei

Nucleus	Percentage of CENH3
1	50.8
2	65.2
3	63.4
4	72.2
5	61.0
6	73.0
7	52.5
8	56.7
9	71.7
10	61.9
11	51.3
12	57.4
13	66.4
14	76.0
15	72.2
16	63.8
**Mean**	**59.5**
**SD**	**8.45**

Supplementary Table 5 Sequence IDs of the analyzed kinetochore proteins in the gene annotation of *Cha. luteum* and *Chi. japonica*

Proteins	*Cha. luteum*	*Chi. japonica*
CENH3	CENH3_Clu_15996.1	CENH3_Chio_7125.1
CENPC	CENPC_Clu_10533	CENPC_Chio_20041.1
	CENPC_Clu_13870.1	CENPC_Chio_23169.1
CENPS	CENPS_Clu_19862.1	CENPS_Chio_6306.1
CENPX	CENPX_Clu_19625.1	CENPX_Chio_6588.1
CENPO	CENPO_Clu_114.1	CENPO_Chio_17369.1
	CENPO_Clu_5032.1	
KNL1	KNL1_Clu_scaffold_5_32848252-32785769.m1	KNL1_Chio_7565.1
		KNL1_Chio_7566.1
		KNL1_Chio_7578.1
		KNL1_Chio_chr12_43561683-43529121.m1
ZWINT1	ZWINT1_Clu_12306.1	ZWINT1_Chio_21569.1
DSN1	DSN1_Clu_12403.1	DSN1_Chio_21647.1
MIS12	MIS12_Clu_9836.1	MIS12_Chio_288.1
NNF1	NNF1_Clu_23982.1	NNF1_Chio_18329.1
NSL1	NSL1_Clu_14614.1	NSL1_Chio_8932.1
	NSL1_Clu_14619.1	
NDC80	NDC80_Clu_11375.1	NDC80_Chio_1734.1
NUF2	NUF2_Clu_22765.1	NUF2_Chio_16151.1
SPC24	SPC24_Clu_6787	SPC24_Chio_4726.1
SPC25	SPC25_Clu_21833.1	SPC25_Chio_12421.1
KNL2	missing	Chio_scaffold_281_255016-245535.m1.p1
		Chio_scaffold_281_255016-245535.m2.p1
		Chio_scaffold_281_255006-252017.m1.p1
		Chio_scaffold_281_255006-252017.m2.p1
NASP	NASP_Clu_8365.1	NASP_Chio_24891.1
		NASP_Chio_24922.1
BMF1	BMF1_Clu_2773.1	BMF1_Chio_13305.1
BMF2	BMF2_Clu_21187.1	BMF2_Chio_19299.1
BMF3	BMF3_Clu_17198.m1	BMF3_Chio_6970.1

BUB3-1/2	BUB3-1_Clu_10898.m1	BUB3_Chio_24588.1
BUB3-3	BUB3-3_Clu_3346	BUB3_Chio_14034.1
MAD1	MAD1_Clu_22666.1	MAD1_Chio_11528.1
MAD2	MAD2_Clu_22531.1	MAD2_Chio_11701.1
MPS1	MPS1_Clu_16746.1	MPS1_Chio_7400.1
AURORA	AURORA_Clu_13708.1	AURORA_Chio_2371.1
	AURORA_Clu_5979.1	AURORA_Chio_22982.1-fs
	AURORA_Clu_3267.1	AURORA_Chio_8142.1
	AURORA_Clu_15903.1	AURORA_Chio_13947.1
BOREALIN	BOREALIN_Clu_4901.1	BOREALIN_Chio_20124.1
INCENP	INCENP_Clu_5148m1	INCENP_Chio_19841.1
SURVIVIN	SURVIVIN_Clu_scaffold_10_19259032-19260258.m1	SURVIVIN_Chio_14439.1

*Genome assembly and gene annotation are available at Zenodo [https://zenodo.org/records/15182433].

Supplementary Table 6 NCBI accession numbers and sources of CENH3 and H3 protein sequences

Plant histone CENH3/ H3	NCBI accession number or source
Chamaelirium luteum CENH3	current study
Chionographis japonica CENH3	(Kuo et al., 2023)[1]
Arabidopsis thaliana H3	AAA32809
Oryza sativa H3	ADI87407
Aegilops tauschii	AKM28569
Allium cepa	BAL45432
Allium sativum	BAL45430
Allium tuberosum	BAL45431
Apostasia shenzhenica	PKA51165
Arabidopsis lyrata	AAT96392
Arabidopsis thaliana	AAL86775
Brassica nigra	ACZ04978
Brassica rapa	NP 001288957
Carex caryophyllea	QGW49120
Carex humilis	QGW49119
Cucumis sativus	XP 011659153
Cucurbita moschata	XP 022959605
Cuscuta campestris A	QGY64362
Cuscuta campestris B	QGY64361
Cuscuta europaea 1A	QGY64356
Cuscuta europaea 2	QGY64360
Cyperus fuscus	QGW49108
Cyperus textilis	QGW49107
Daucus glochidiatus	AID21731
Daucus pusillus	AID21730
Elaeis guineensis X1	XP 019708718
Elaeis guineensis X2	XP 010931498
Luzula elegans 1	AOR06534
Luzula elegans 2	AOR06535
Luzula nivea	ADM18965
Musa acuminata	AKI32604
Musa balbisiana	AMH40810
Nicotiana sylvestris	NP 001289496
Nicotiana tomentosiformis	NP 001289450
Oryza alta	ACX30889
Oryza australiensis	ACX30893
Phalaenopsis equestris X1	XP 020572267
Phalaenopsis equestris X2	XP 020572268
Phoenix dactylifera	XP 008792454
Prionium serratum	Baez et al., 2020
Raphanus sativus	BAF49733
Rhynchospora pubera 1	ALF04639
Rhynchospora pubera 2	ALF04640
Secale sylvestre alpha	AUN88454
Secale sylvestre beta	AUN88469
Solanum lycopersicum	XP 010326926
Solanum tuberosum	XP 006339687

Triticum aestivum	AEH95350
Vitis vinifera 1	XP 010661899
Vitis vinifera 2	XP 002281073
Zea mays	NP 001105520

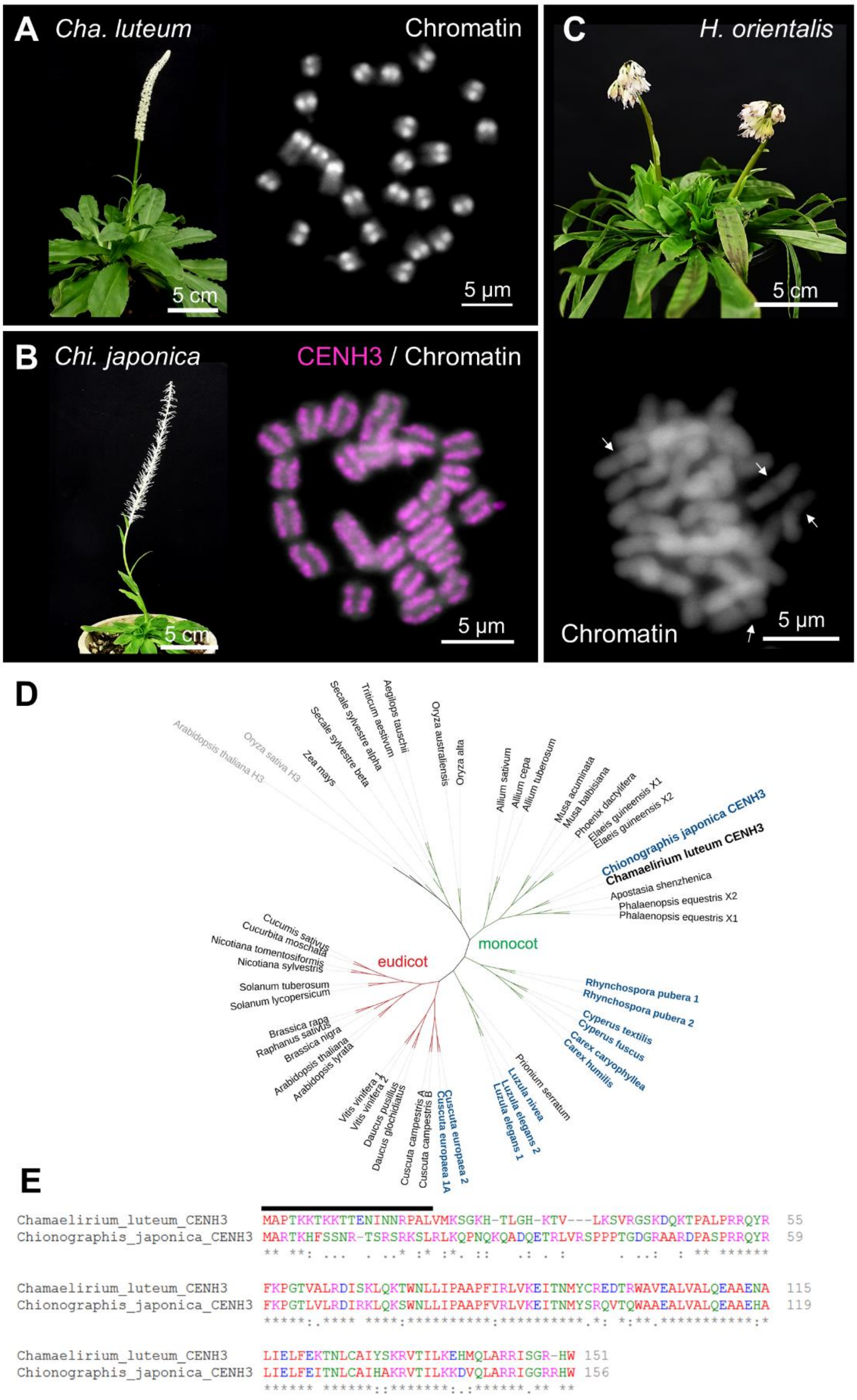

Supplementary Fig. 1 Diversity of chromosome and centromere structure in three Melanthiaceae species and identification of *Chamaelirium luteum* CENH3. (A) Flowering *Cha. luteum* features large heterochromatic monocentromeres that protrude at mitotic metaphase. The monocentromere-typical primary construction is missing in this species. (B) Flowering holocentric *Chionographis japonica* possesses

chromosome-wide CENH3-immunosignals at the two peripheries of metaphase chromosomes (magenta). (C) Flowering *Heloniopsis orientalis* has monocentric chromosomes with a typical primary constriction (arrows). Chromosomes were counterstained with DAPI. (D) Phylogenetic tree of mono- and eudicot CENH3 proteins. CENH3 of monocentric *Cha. luteum* grouped with the CENH3 of holocentric *Chi. japonica.* Histone H3 of *Arabidopsis thaliana* and *Oryza sativa* was used as an outgroup. (E) Amino acid sequence alignment of CENH3s from *Cha. luteum* and *Chi. japonica*. The peptide sequence used for the generation of the *Cha. luteum*-specific anti-CENH3 antibody is indicated with a black line. At least two independent experiments were carried out to confirm the reproducibility of the labeling patterns.

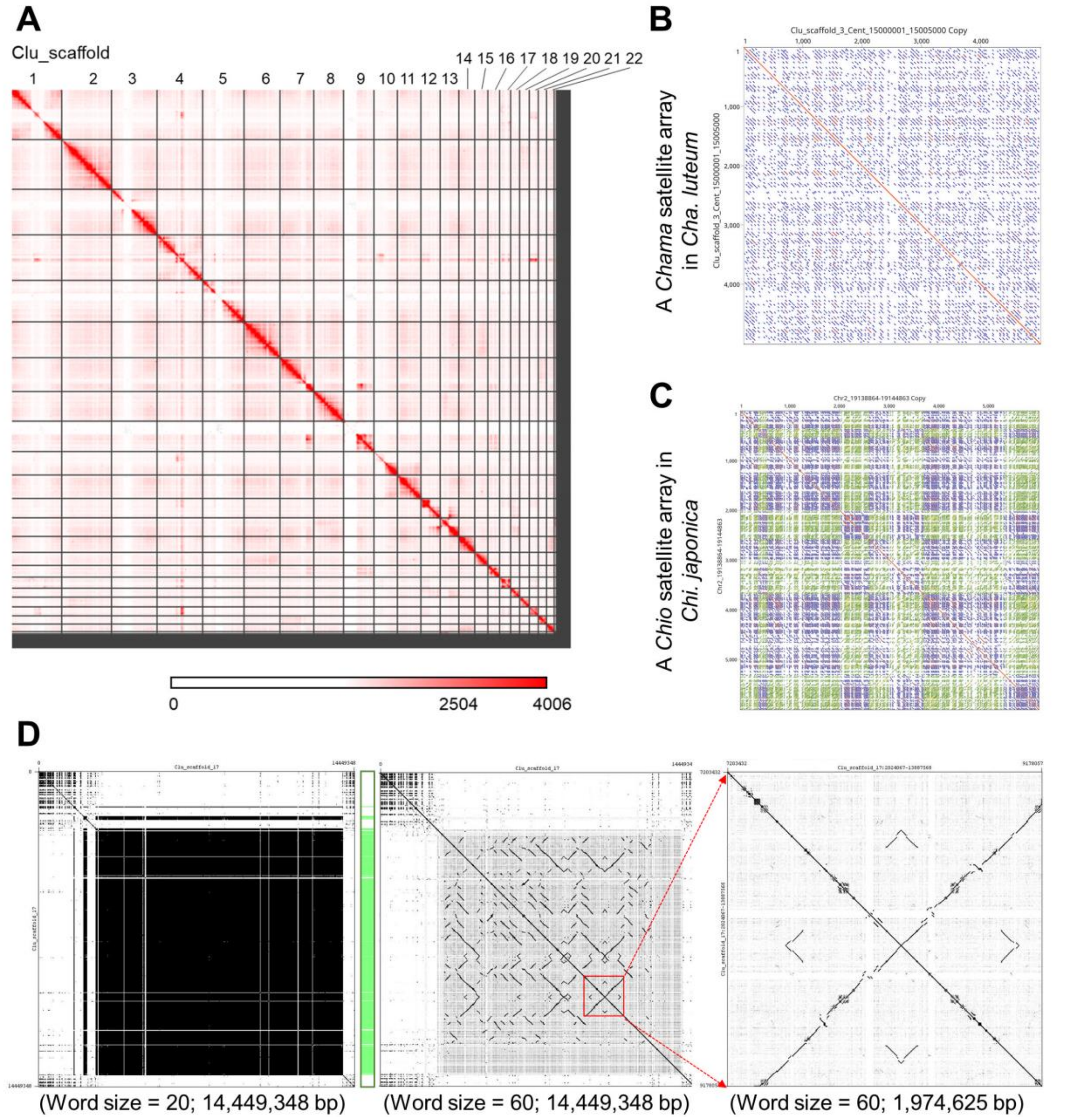

Supplementary Fig. 2 Hi-C map of *Cha. luteum* and contrasting centromere array organization in *Cha. luteum* and *Chi. japonica*. (A) Hi-C scaffolding of *Cha. luteum* contigs result in 22 scaffolds longer than 10 Mb (10–68 Mb) (Supplementary Table 2). The color bar represents the contact frequencies, which are indicated by the number of links at a 5-Mb resolution. (B) The centromeric *Chama* monomers in *Cha. luteum* are arranged in the same orientation in a ~5-kb satellite repeat array, while (C) in *Chi. japonica*, the orientation in a ~6-kb centromeric *Chio* satellite array is different. Blue and green lines represent forward- and reverse-strand similarity, respectively. Red lines indicate regions of 100% sequence identity. (D) Dotplot analysis of Clu_scaffold_17. Under the relaxed condition (word size 20), *Chama* arrays appear highly homogeneous (left). Under the strict condition (word size 60, matching the 60-

bp *Chama* monomer), the analysis reveals the presence of higher-order repeat structure (middle). The green bar indicates the distribution of *Chama* repeats. Furthermore, many *Chama* arrays maintain a consistent orientation across several hundred kilobases (middle), with some extending over regions larger than 1 Mb (right). The large-scale dotplot analysis was performed using Gepard v. 2.1 [2].

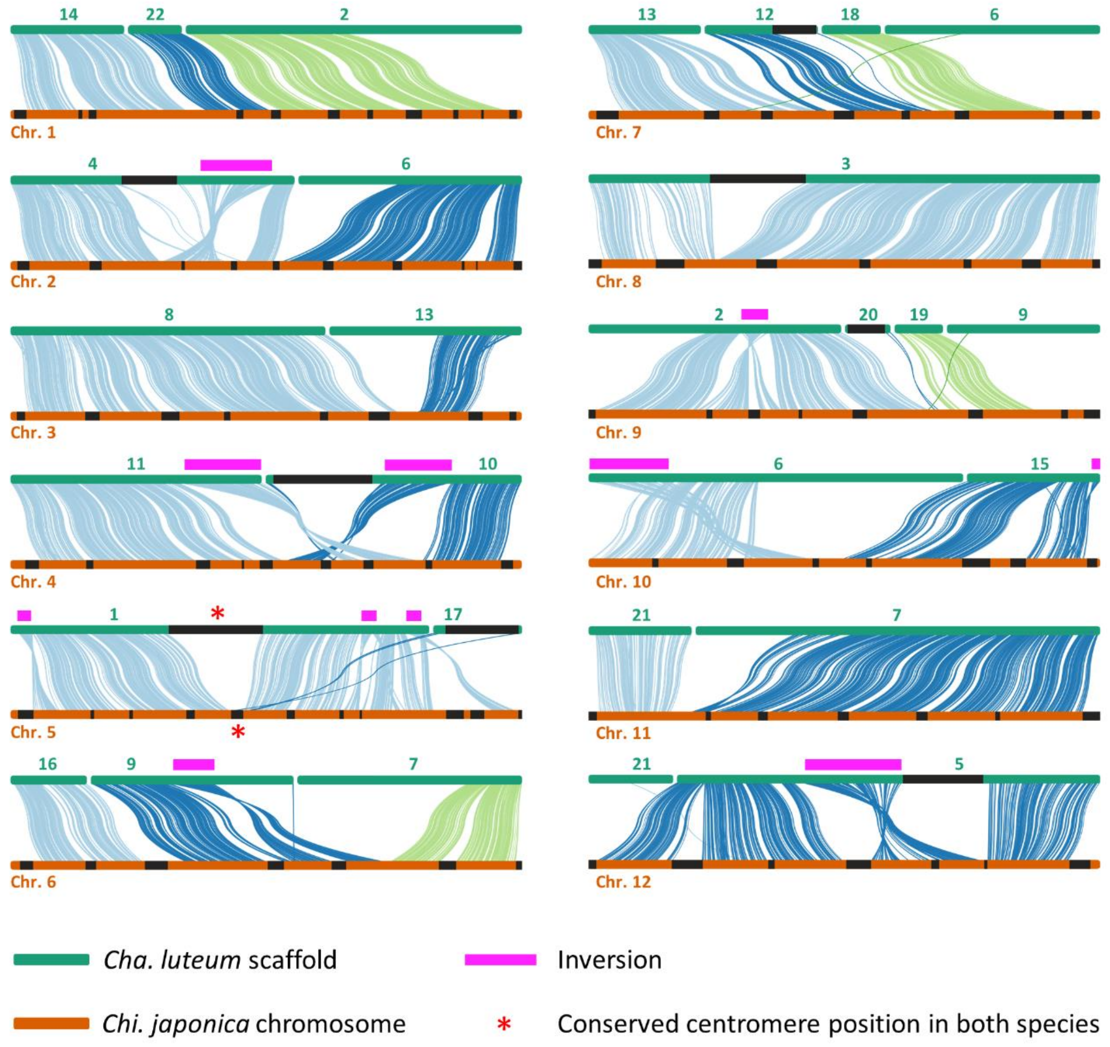

Supplementary Fig. 3 Holocentric *Chi. japonica* and monocentric *Cha. luteum* share broad-scale genome synteny, except for their centromeres. Visualization of syntenic orthologs of coding genes in the assembled top 22 scaffolds of *Cha. luteum* (green) and the 12 chromosome-level pseudomolecules of *Chi. japonica* (orange). The chromosome-level arrangement of orthologs between scaffold 3 of *Cha. luteum* and chromosome 8 of *Chi. japonica* is identical. In addition to chromosome-sized syntenic regions, 11 large-scale inversions (magenta lines) and four inter-chromosomal translocations (scaffolds 2, 6, 7, and 13 of *Cha. luteum*) were identified. The conserved centromere positions in both species are marked by asterisks.

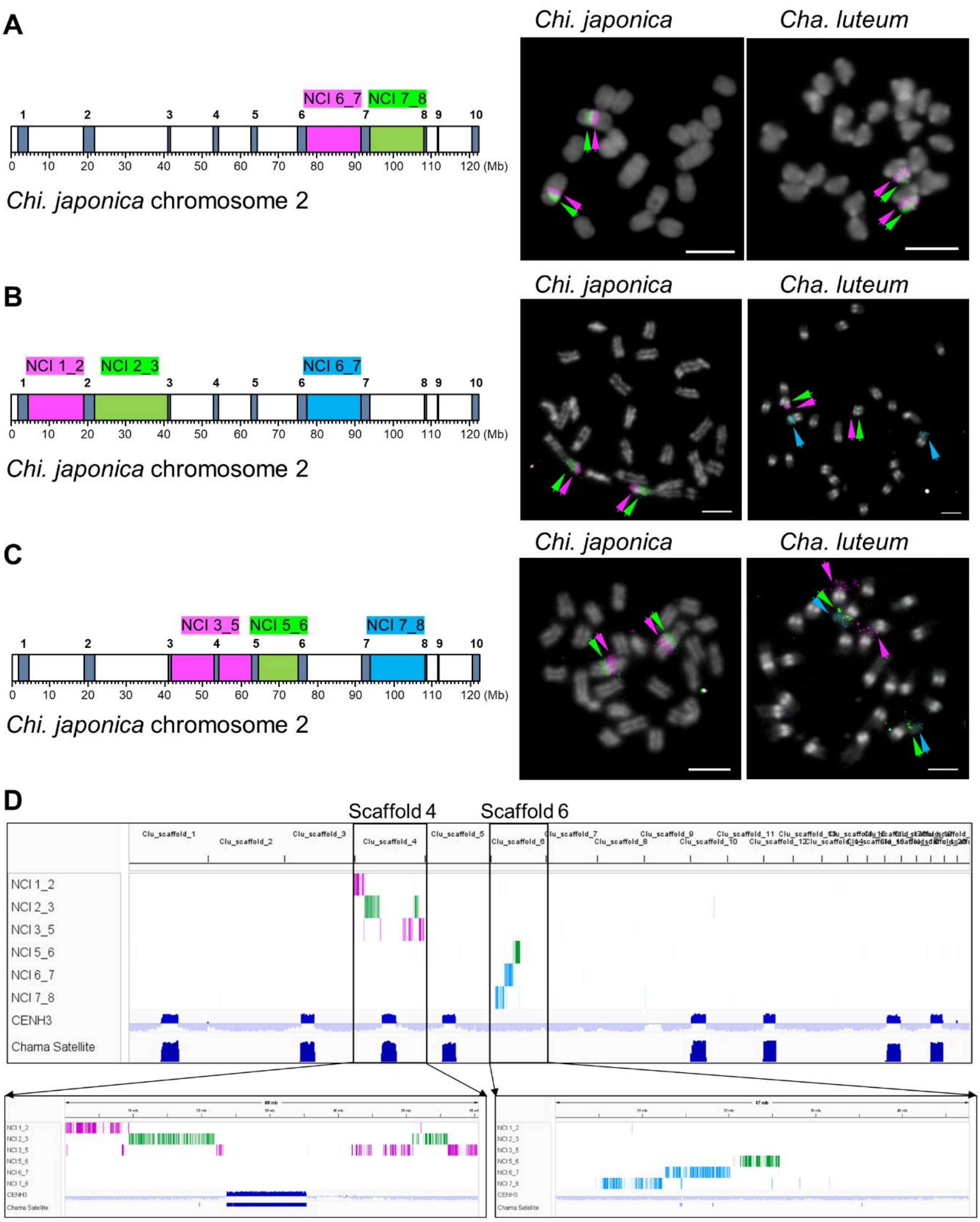

Supplementary Fig. 4 Comparative FISH mapping of the *Chi. japonica* chromosome 2-specific oligo-FISH probes on mitotic chromosomes of *Chi. japonica* and *Cha. luteum*. (A) Application of the multicolor non-centromeric interval (NCI)-specific oligo-FISH painting probes NCI 6_7 (magenta) and NCI 7_8 (green). (B) Application of the oligo painting probes NCI 1_2 (magenta), NCI 2_3 (green), and NCI 6_7 (blue). (C) Application of the oligo painting probes NCI 3_5 (magenta), NCI 5_6 (green), and NCI 7_8 (blue). Schemata show the chromosomal position and color of the probes along

Chi. japonica chromosome 2. Colored arrows indicate the signals of the corresponding probes shown in the schemata. (D) *In silico* mapping of the six *Chi. japonica* chromosome 2-specific oligo probes onto the scaffolds 4 and 6 of *Cha. luteum*, which correspond to the three chromosome arms of two *Cha. luteum* chromosome pairs. Chromosomes were counterstained with DAPI. Scale bars = 5 μm. At least two independent experiments were carried out to confirm the reproducibility of the labeling patterns.

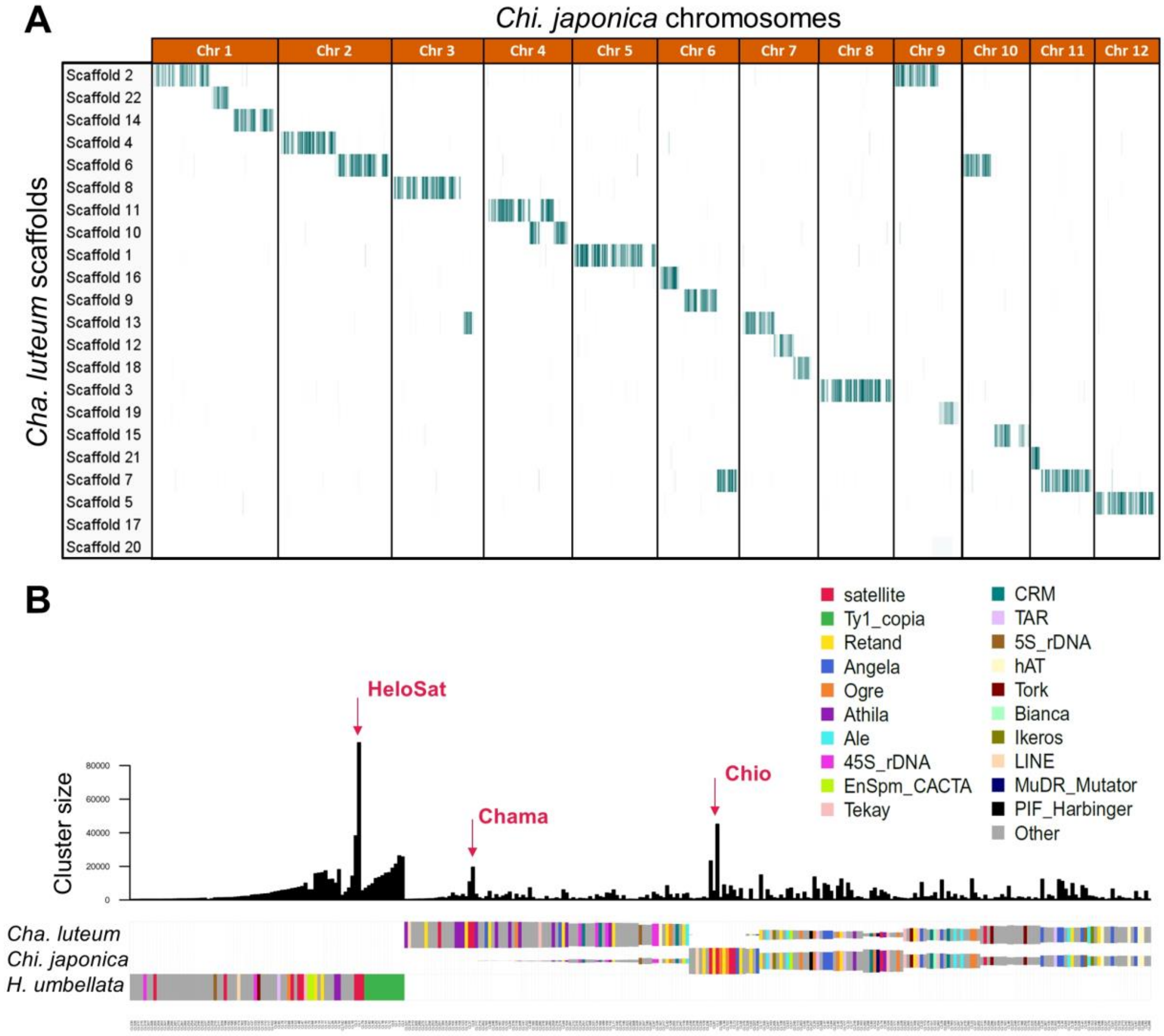

Supplementary Fig. 5 Comparative sequence analyses among Melanthiaceae species. (A) *In silico* comparative mapping of 12 *Chi. japonica*-based chromosome-wide oligo pools to the top 22 scaffolds of *Cha. luteum* confirmed high chromosomal collinearity between the two genomes and revealed no evidence of chromosome duplication. (B) Comparative repeat analysis using RepeatExplorer revealed no shared high-copy repeats between *Heloniopsis umbellata* and either *Cha. luteum* or *Chi. japonica.* Arrows indicate the position of the known, highly abundant satellite repeats *HeloSat* [3], *Chama* (this study) and *Chio* [1]. The bar plot represents the abundance of each cluster. The annotation of clusters is shown in different colors.

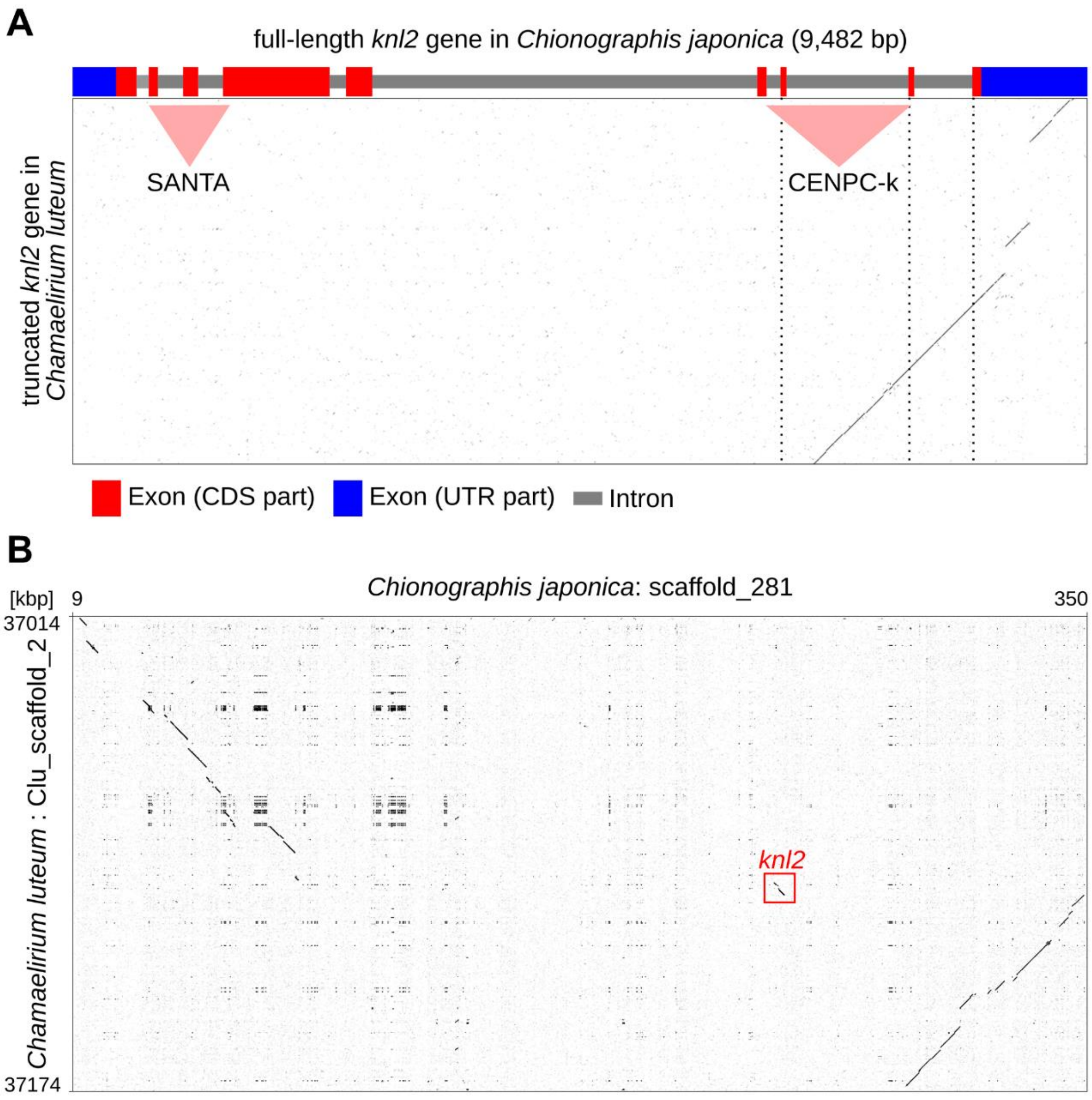

Supplementary Fig. 6 Loss of the *KNL2* gene in *Cha. luteum*. (A) A detailed dot plot comparison of the *KNL2* genes shows that most of the *KNL2* gene has been lost in *Cha. luteum*. Light red triangles mark the positions encoding the SANTA domain and the CENPC-k motif, indicating a complete loss of the SANTA-coding domain. Although a portion of the CENPC-k coding domain is retained in the truncated gene, it corresponds to the last nine amino acids at the C-terminus and could not be translated into a protein due to the absence of a start codon in the upstream region. (B) Dot plot comparison of orthologous loci between *Cha. luteum* and *Chi. japonica*. The position of the *KNL2* gene is marked by the red rectangle.

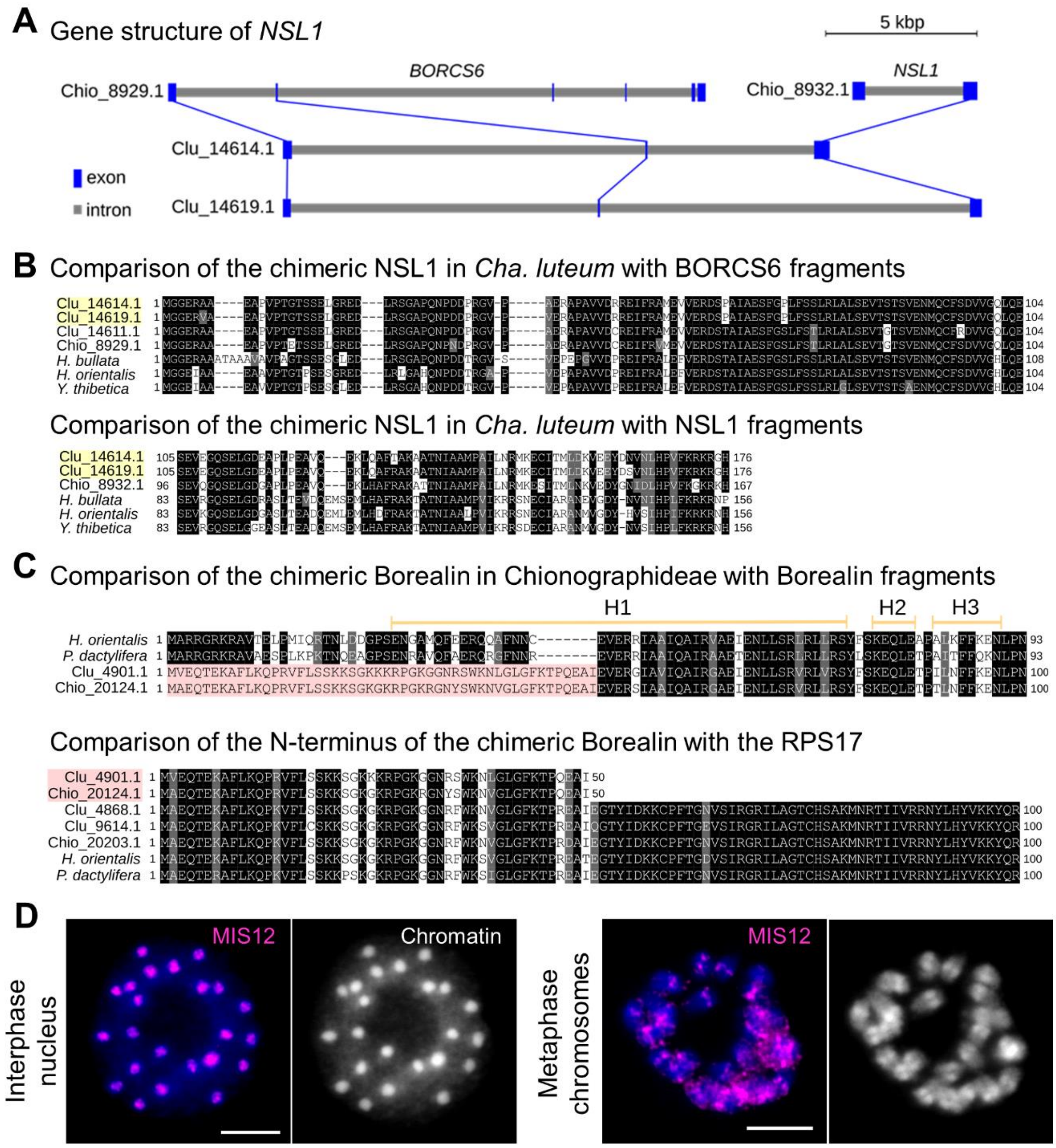

Supplementary Fig. 7 Chimeric origin of NSL1 in *Cha. luteum* and Borealin in both *Cha. luteum* and *Chi. japonica* and immunodetection of MIS12 in *Cha. luteum.* (A) In *Cha. luteum*, the *NSL1* gene has undergone significant changes through recombination with the *BORCS6* gene. The changes in these genes are shown by comparison with full-length homologous genes, *NSL1* (Chio_8932.1) and *BORCS6* (Chio_8929.1), identified in *Chi. japonica*. Note that *Cha. luteum* has two copies of the recombined *NSL1* gene (Clu_14614.1 and Clu_14619.1). (B) Comparison of the N- and C-termini of the chimeric NSL1 protein sequences in *Cha. luteum* with the corresponding parts of BORCS6 and NSL1 homologous proteins in *Chi. japonica* and three other Melanthiaceae species (*Helonias bullata, Heloniopsis orientalis*, and *Ypsilandra thibetica*). The recombinant NSL1 protein sequences in *Cha. luteum* are

highlighted in yellow. (C) The N-terminus of Borealin in both *Cha. luteum* and *Chi. japonica* is replaced by the N-terminal fragment of ribosomal protein S17 (RPS17). Alignments of the protein sequences at the N-termini of the recombinant Borealin proteins with the corresponding parts of the intact Borealin and RPS17 in *H. orientalis* and *Phoenix dactylifera*. (D) Recruitment of MIS12 to centromeres of *Cha. luteum* remains unaffected. Immunolabelling of MIS12 in an interphase nucleus and on metaphase chromosomes of *Cha. luteum* using the *Chi. japonica*-specific anti-MIS12 antibody. Chromatin was counterstained with DAPI. Scale bars = 5 µm. At least two independent experiments were carried out to confirm the reproducibility of the labeling patterns.

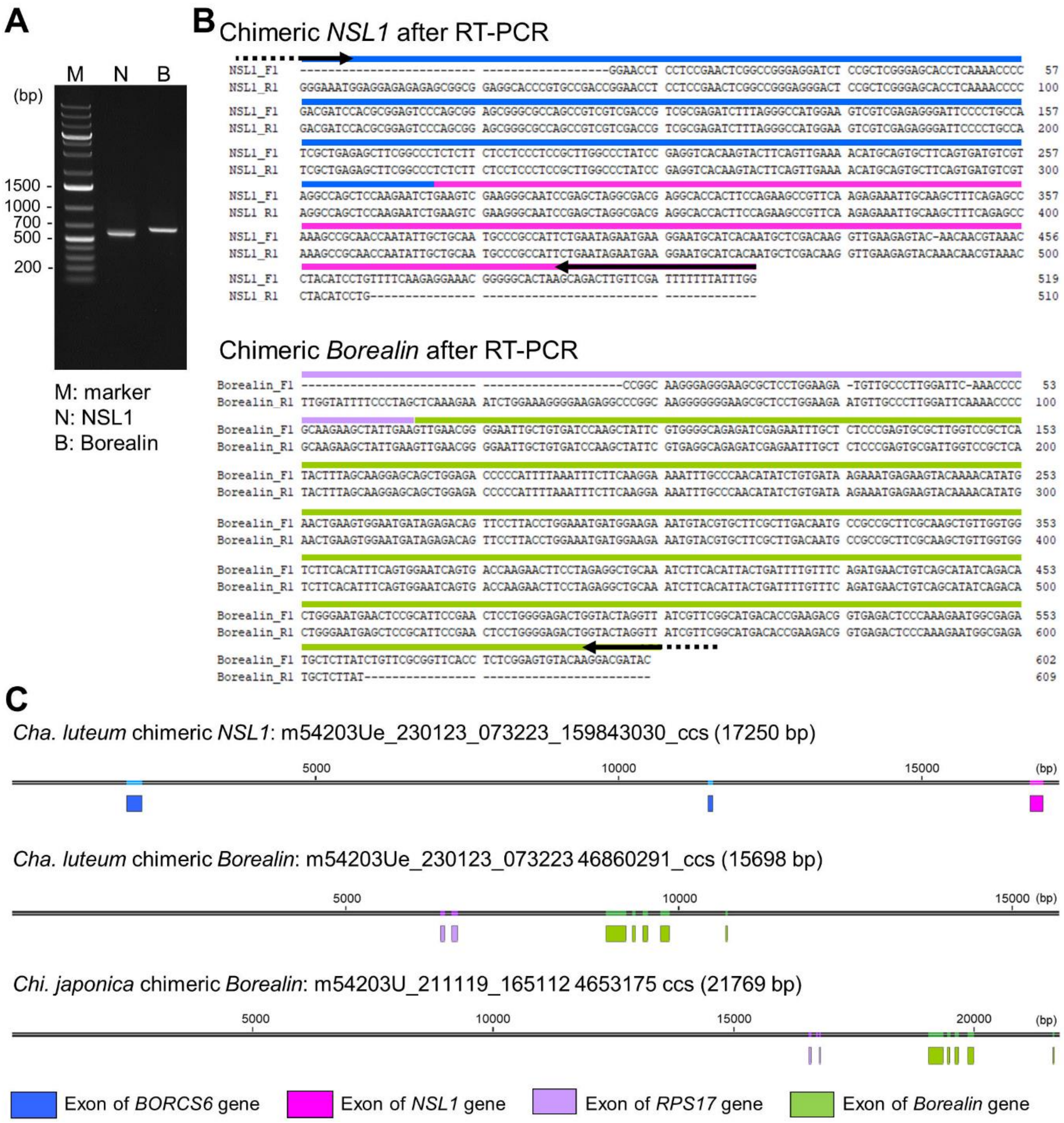

Supplementary Fig. 8 Verification of the chimeric *NSL1* and *Borealin* genes at the expression and genomic levels. (A) RT-PCR amplification of *NSL1* (565 bp) and *Borealin* (655 bp) from *Cha. luteum* produced fragments of the expected sizes based on transcriptome analysis. (B) Sanger sequence alignment of RT-PCR products confirmed the fusion junctions. Gene fragments are indicated by colored lines: *BORCS6* (blue), *NSL1* (magenta), *RPS17* (light purple), and *Borealin* (green). Black arrows represent the employed RT-PCR primers. (C) Full-length genomic structure of the chimeric *NSL1* and *Borealin* genes was captured in single PacBio reads from *Cha. luteum* and *Chi. japonica*, supporting their genuine chimeric origin.

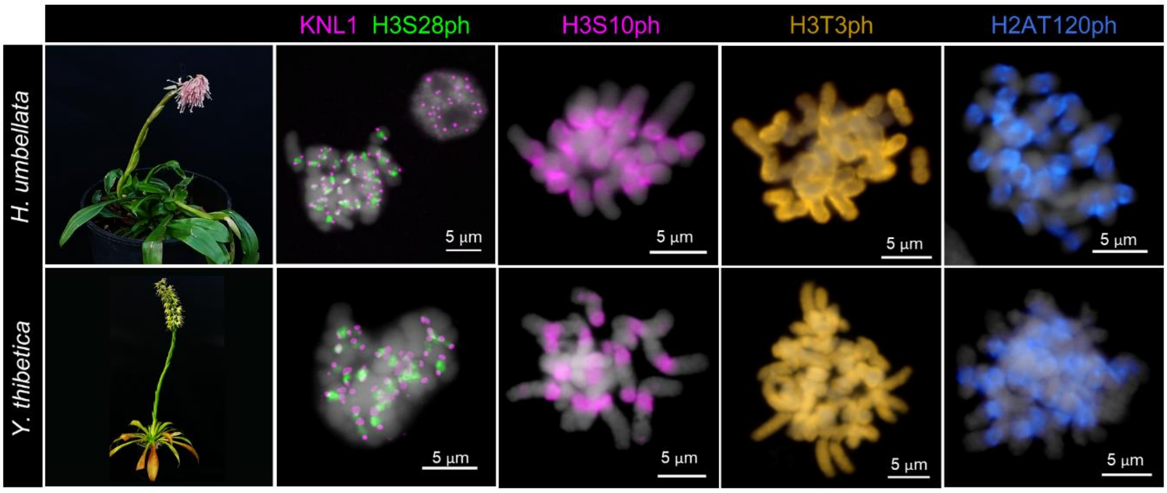

Supplementary Fig. 9 The Heloniadeae species possess monocentromeres with monocentric-typical histone phosphorylation patterns. Flowering *Heloniopsis umbellata* and *Ypsilandra thibetica* plants. Immunostaining of mitotic chromosomes using the kinetochore antibody KNL1 (magenta) and the cell cycle-dependent histone marks H3S28ph (green), H3S10ph (magenta), H3T3ph (yellow), and H2AT120ph (blue) in both species. Chromatin was counterstained with DAPI. At least two independent experiments were carried out to confirm the reproducibility of the labeling patterns.

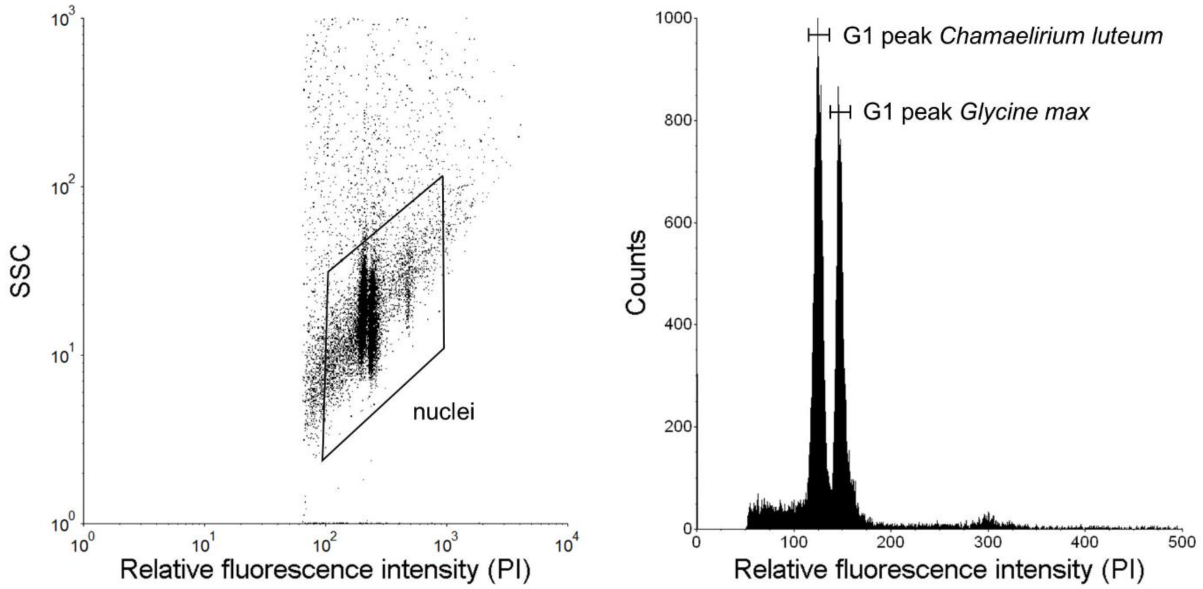

Supplementary Fig. 10 Gating strategy for genome size measurement of *Cha. luteum* using flow cytometry. Nuclei were separated from cellular debris by plotting the log-scale relative fluorescence intensity of the propidium iodide (PI) staining against the log-scale side scatter (SSC) signal using a green laser (532 nm) for excitation. The G1 peaks of *Cha. luteum* and the internal reference standard (*Glycine max*) were identified in the corresponding histogram displaying the lin-scale PI relative fluorescence intensity of the nuclear fraction.

Supplementary References

1. Kuo Y-T*, et al.* Holocentromeres can consist of merely a few megabase-sized satellite arrays. *Nat Commun* **14**, 3502 (2023).

2. Krumsiek J, Arnold R, Rattei T. Gepard: a rapid and sensitive tool for creating dotplots on genome scale. *Bioinformatics* **23**, 1026-1028 (2007).

3. Pellicer J, Fernandez P, Fay MF, Michalkova E, Leitch IJ. Genome Size Doubling Arises From the Differential Repetitive DNA Dynamics in the Genus Heloniopsis (Melanthiaceae). *Front Genet* **12**, 726211 (2021).
