# Supplementary material for: Kinetochore mutations and histone phosphorylation pattern changes accompany holo- and macro-monocentromere evolution
Source: Nat Commun. 2025 Dec 20;16:11332. doi: 10.1038/s41467-025-67524-8 (PMC12722358; doi:10.1038/s41467-025-67524-8)
Supplement: Supplementary file 2 — Description of Additional Supplementary Files [file 41467_2025_67524_MOESM2_ESM.pdf]

Description of Additional Supplementary Files

File Name: Supplementary Movie 1
Description: Immunostaining of CENH3 (magenta) and spindle microtubules (green) in metaphase chromosomes of Cha. luteum.
