# Supplementary material for: Kinetochore mutations and histone phosphorylation pattern changes accompany holo- and macro-monocentromere evolution
Source: Nat Commun. 2025 Dec 20;16:11332. doi: 10.1038/s41467-025-67524-8 (PMC12722358; doi:10.1038/s41467-025-67524-8)
Supplement: Supplementary file 4 — Reporting Summary [file 41467_2025_67524_MOESM4_ESM.pdf]

nature portfolio

Corresponding author(s): Yi-Tzu Kuo and Andreas Houben

Last updated by author(s): 2025.11.20

Reporting Summary

Nature Portfolio wishes to improve the reproducibility of the work that we publish. This form provides structure for consistency and transparency in reporting. For further information on Nature Portfolio policies, see our Editorial Policies and the Editorial Policy Checklist.

Statistics

For all statistical analyses, confirm that the following items are present in the figure legend, table legend, main text, or Methods section.

n/a	Confirmed	
☐	☒	The exact sample size (*n*) for each experimental group/condition, given as a discrete number and unit of measurement
☐	☒	A statement on whether measurements were taken from distinct samples or whether the same sample was measured repeatedly
☒	☐	The statistical test(s) used AND whether they are one- or two-sided<br>*Only common tests should be described solely by name; describe more complex techniques in the Methods section.*
☒	☐	A description of all covariates tested
☒	☐	A description of any assumptions or corrections, such as tests of normality and adjustment for multiple comparisons
☐	☒	A full description of the statistical parameters including central tendency (e.g. means) or other basic estimates (e.g. regression coefficient) AND variation (e.g. standard deviation) or associated estimates of uncertainty (e.g. confidence intervals)
☒	☐	For null hypothesis testing, the test statistic (e.g. *F*, *t*, *r*) with confidence intervals, effect sizes, degrees of freedom and *P* value noted<br>*Give P values as exact values whenever suitable.*
☒	☐	For Bayesian analysis, information on the choice of priors and Markov chain Monte Carlo settings
☒	☐	For hierarchical and complex designs, identification of the appropriate level for tests and full reporting of outcomes
☒	☐	Estimates of effect sizes (e.g. Cohen's *d*, Pearson's *r*), indicating how they were calculated

Our web collection on statistics for biologists contains articles on many of the points above.

Software and code

Policy information about availability of computer code

Data collection	Genome size measurement was performed using a Sysmex CyFlow Space flow cytometer (Sysmex-Partec, Germany). The Epifluorescence microscope BX61 (Olympus, Germany), Elyra PS.1 microscope (Carl Zeiss), and Transmission Electron Microscope Tecnai Sphera G2 120 kV (FEI) were used to take microscopic images. Sequencing was performed using the Pacific Biosciences Revio (HiFi CCS), NovaSeq6000 (Illumina Inc., USA), and DNBSEQ (BGI, China) device.
Data analysis	Genome size measurement: FloMax Operating and Analysis Software for Flow Cytometry Particle Analysing Systems, Version 2.82; The images analyzed were performed using the Zeiss ZENBlack (Carl Zeiss GmbH, Germany), Imaris 9.7 (Bitplane, UK), Adobe Photoshop 6.0 (Adobe), Image J softwares. Hifiasm Version v0.19.3-r572 (https://github.com/chhylp123/hifiasm) was used for genome assembly; Quast Version2.3 (https://github.com/ablab/quast) was used for contig statistics. BUSCO v4.1.2 was used to assess the genome assembly. purge_dups v 1.2.5 was used to identify and remove duplicated sequence segments in the primary assembly. The Arima Genomics mapping pipeline (https://github.com/ArimaGenomics/mapping_pipeline) was used to process the Hi-C data; YaHS (v1.2a.2) was used for scaffolding. Hi-C contact maps and manual curation were accomplished by the bash scripts provided (https://github.com/c-zhou/yahs) and visualized using Juicebox (https://github.com/aidenlab/Juicebox). HISAT2 (v2.2.1) and StringTie (v2.1.1), gffread (v0.12.6), TransDecoder (v5.5.0), and BRAKER3 pipeline were used for genome-directed transcriptome assembly and gene annotation. The Python scripts for genome and synteny analyses are available in BitBuckket (https://bitbucket.org/ipk-csf/chamaelirium2chiographis/). NGenomeSyn (v1.41) was used to visualize the genome synteny. Trinity (v2.4.0) was used for de novo assembly of RNA-seq datasets; TransDecoder (v5.5.0) was used to identify putative coding regions. The est2genome (https://www.bioinformatics.nl/cgi-bin/emboss/est2genome) and genewise (https://www.ebi.ac.uk/jdispatcher/psa/genewise) were used for gene annotation in kinetochore analysis. Genome repetitive analysis was carried out using FastQC Galaxy Version 0.72, RepeatExplorer2 Galaxy Version 2.3.8.1, and TAREAN Galaxy Version 2.3.8.1 implemented in Galaxy server (https://repeatexplorer-elixir.cerit-sc.cz/galaxy/). ChIPseq datasets were analyzed using ChIP-Seq Mapper Galaxy Version 1.1.1.4 (https://repeatexplorer-elixir.cerit-sc.cz/galaxy/), Bowtie2 Galaxy Version 2.5.3 (https://galaxy.ipk-gatersleben.de/), MACS Version 3.0.1 (https://

github.com/macs3-project/MACS), and deeptools Galaxy Version 3.5.4. Genome browser tracks were produced using pyGenomeTracks Galaxy Version 3.8 (https://github.com/deeptools/pyGenomeTracks). ATAC-seq were analyzed using fastp (v0.20.0) for adapter trimming, BWA-MEM (v0.7.17) for genome alignment, SAMtools (v1.16.1) for removing duplicate and multiple-mapping reads, MACS (v3.0.0) and for peak calling, deepTools bamCoverage (v3.5.1) for converting BAM to BigWig format. Chorus2 Version 2.0.1 (https://github.com/zhangtaolab/Chorus2) was used for oligo probe design.

For manuscripts utilizing custom algorithms or software that are central to the research but not yet described in published literature, software must be made available to editors and reviewers. We strongly encourage code deposition in a community repository (e.g. GitHub). See the Nature Portfolio guidelines for submitting code & software for further information.

Data

Policy information about availability of data

All manuscripts must include a data availability statement. This statement should provide the following information, where applicable:

- Accession codes, unique identifiers, or web links for publicly available datasets
- A description of any restrictions on data availability
- For clinical datasets or third party data, please ensure that the statement adheres to our policy

The whole-genome sequencing and RNA-seq datasets generated for this study can be found at EMBL-ENA under the project IDs PRJEB82607 and PRJEB82608, respectively. The datasets of ChIP-Seq (Project ID: PRJNA1201173, accession GSE285103) and ATAC-Seq (Project ID: PRJNA1201177, accession GSE285102) were deposited in the NCBI GEO database. The gene annotation, syntenic genes, and sequences of oligo-FISH painting probes and proteins are available in Zenodo (https://zenodo.org/records/15182433).

Research involving human participants, their data, or biological material

Policy information about studies with human participants or human data. See also policy information about sex, gender (identity/presentation), and sexual orientation and race, ethnicity and racism.

Reporting on sex and gender	N/A
Reporting on race, ethnicity, or other socially relevant groupings	N/A
Population characteristics	N/A
Recruitment	N/A
Ethics oversight	N/A

Note that full information on the approval of the study protocol must also be provided in the manuscript.

Field-specific reporting

Please select the one below that is the best fit for your research. If you are not sure, read the appropriate sections before making your selection.

[x] Life sciences [ ] Behavioural & social sciences [ ] Ecological, evolutionary & environmental sciences

For a reference copy of the document with all sections, see nature.com/documents/nr-reporting-summary-flat.pdf

Life sciences study design

All studies must disclose on these points even when the disclosure is negative.

Sample size	We decided the sample size based on available literatures in the field, our own experience in previous studies, and requirement for corresponding protocols. The sample sizes used for all experiments provided sufficient resolving power.
Data exclusions	No data was excluded from the analysis.
Replication	The number of replications is indicated in the section Methods and Figure legends. For fluorescence in situ hybridization and immunostaining, at least two independent experiments were carried out to confirm the reproducibility of the labeling patterns.
Randomization	A randomization is not relevant for this study because no genotype or treatment were compared with each other. However, the tissues for cytogenetic and sequencing experiments were randomly collected from different plant individuals grown under the same condition in a greenhouse. Only for genome assembly, we used the HMW DNA from a single Chamaelirium luteum plant for long-read sequencing, to avoid problems due to the highly heterozygous genome of this species.
Blinding	All the experiments were performed without prior knowledge of the final outcome, and therefore blinding was not applied.

Reporting for specific materials, systems and methods

We require information from authors about some types of materials, experimental systems and methods used in many studies. Here, indicate whether each material, system or method listed is relevant to your study. If you are not sure if a list item applies to your research, read the appropriate section before selecting a response.

Materials & experimental systems

n/a	Involved in the study
☐	☒ Antibodies
☒	☐ Eukaryotic cell lines
☒	☐ Palaeontology and archaeology
☒	☐ Animals and other organisms
☒	☐ Clinical data
☒	☐ Dual use research of concern
☐	☒ Plants

Methods

n/a	Involved in the study
☐	☒ ChIP-seq
☐	☒ Flow cytometry
☒	☐ MRI-based neuroimaging

Antibodies

Antibodies used

Customized antibodies:
rabbit anti-ClCENH3 (current study)
rabbit anti-Chi. japonica MIS12 (Kuo et al., 2023; https://doi.org/10.1038/s41467-023-38922-7)
rabbit anti-Chi. japonica NDC80 (Kuo et al., 2023; https://doi.org/10.1038/s41467-023-38922-7)
rabbit anti-Cuscuta europaea KNL1 (Neumann et al., 2023; https://doi.org/10.1073/pnas.2300877120)

Commercially available antibodies:
mouse anti-alpha-tubulin (Sigma-Aldrich, USA, cat. no. T9026-2, dilution 1:300)
rabbit anti-histone H3K4me2 (abcam, UK, cat. no. ab7766, dilution 1:300)
mouse anti-histone H3K9me2 (abcam, UK, cat. no. ab1220, dilution 1:200)
mouse anti-histone H3S10ph (abcam, UK, cat. no. ab14955, dilution 1:1000)
rat anti-histone H3S28ph (Sigma-Aldrich, USA, cat. no. H9908, dilution 1:1000)
mouse anti-H3T3ph (Sigma-Aldrich, USA, cat. no. 07-424, dilution 1:1000)
rabbit anti-H2AT120ph (Active Motif, USA, cat. no. 61196, dilution 1:500)
anti-rabbit rhodamine (Jackson ImmunoResearch, USA, cat. no. 111-295-144, dilution 1:400)
anti-rabbit Alexa488 (Jackson ImmunoResearch, USA, cat. no. 711-545-152, dilution 1:400)
anti-mouse Alexa488 (Jackson ImmunoResearch, USA, cat. no. 715-546-151, dilution 1:400)
anti-rat Alexa488 (Jack ImmunoResearch, USA, cat. no. 112-545-167, dilution 1:400)

Validation

Validation by commercial providers:
mouse anti-alpha-tubulin (Sigma-Aldrich, USA, cat. no. T9026-2), https://www.sigmaaldrich.com/DE/de/product/sigma/t9026?gclid=EAIaIQobChMIgZ3Z0_my_gIVEepRCh2ZzAYvEAAYASAAEgKmOfD_BwE&gclsrc=aw.ds
rabbit anti-histone H3K4me2 (abcam, UK, cat. no. ab7766), https://www.abcam.com/products/primary-antibodies/histone-h3-di-methyl-k4-antibody-chip-grade-ab7766.html
mouse anti-histone H3K9me2 (abcam, UK, cat. no. ab1220), https://www.abcam.com/products/primary-antibodies/histone-h3-di-methyl-k9-antibody-mabcam-1220-chip-grade-ab1220.html
mouse anti-histone H3S10ph (abcam, UK, cat. no. ab14955), https://www.abcam.com/products/primary-antibodies/histone-h3-phospho-s10-antibody-mabcam-14955-ab14955.html
rat anti-histone H3S28ph (Sigma-Aldrich, USA, cat. no. H9908), https://www.sigmaaldrich.com/DE/de/product/sigma/h9908
mouse anti-H3T3ph (Sigma-Aldrich, USA, cat. no. 07-424), https://www.merckmillipore.com/DE/de/product/Anti-phospho-Histone-H3-Thr3-Antibody,MM_NF-07-424?ReferrerURL=https%3A%2F%2Fwww.google.com%2F
rabbit anti-H2AT120ph (Active Motif, USA, cat. no. 61196)
https://www.activemotif.com/catalog/details/61195/histone-h2a-phospho-thr120-antibody-pab-1
anti-rabbit rhodamine (Jack ImmunoResearch, USA, cat. no. 111-295-144), https://www.jacksonimmuno.com/catalog/products/111-295-144
anti-rabbit Alexa488 (Jack ImmunoResearch, USA, cat. no. 711-545-152), https://www.jacksonimmuno.com/catalog/products/711-545-152
anti-mouse Alexa488 (Jack ImmunoResearch, USA, cat. no. 715-546-151), https://www.jacksonimmuno.com/catalog/products/715-546-151
anti-rat Alexa488 (Jack ImmunoResearch, USA, cat. no. 112-545-167), https://www.jacksonimmuno.com/catalog/products/112-545-167

The customized peptide rabbit anti-ClCENH3 antibodies generated by the company LifeTein were validated by peptide ELISA tests. The ELISA information is available upon request. The rabbit anti-Chi. japonica MIS12, rabbit anti-Chi. japonica NDC80, and rabbit anti-Cuscuta europaea KNL1 were validated in the corresponding published literature, and the tested species were included as positive control in the immunostaining experiments.

Dual use research of concern

Policy information about dual use research of concern

Hazards

Could the accidental, deliberate or reckless misuse of agents or technologies generated in the work, or the application of information presented in the manuscript, pose a threat to:

No	Yes	
☒	☐	Public health
☒	☐	National security
☒	☐	Crops and/or livestock
☒	☐	Ecosystems
☒	☐	Any other significant area

Experiments of concern

Does the work involve any of these experiments of concern:

No	Yes	
☒	☐	Demonstrate how to render a vaccine ineffective
☒	☐	Confer resistance to therapeutically useful antibiotics or antiviral agents
☒	☐	Enhance the virulence of a pathogen or render a nonpathogen virulent
☒	☐	Increase transmissibility of a pathogen
☒	☐	Alter the host range of a pathogen
☒	☐	Enable evasion of diagnostic/detection modalities
☒	☐	Enable the weaponization of a biological agent or toxin
☒	☐	Any other potentially harmful combination of experiments and agents

Plants

Seed stocks	Chamaelirium luteum (L.) A. Gray plants used in this study were provided by the Deutsche Homöopathie-Union (DHU), Germany, Chionographis japonica (Willd.) Maxim. plants were obtained from commercial nurseries in Japan, and the Helonias bullata L., Heloniopsis umbellata Baker, Heloniopsis orientalis var. breviscapa, and Ypsilandra thibetica Franch. species were purchased from British nurseries in the UK.
Novel plant genotypes	N/A
Authentication	N/A

ChIP-seq

Data deposition

☒ Confirm that both raw and final processed data have been deposited in a public database such as GEO.

☒ Confirm that you have deposited or provided access to graph files (e.g. BED files) for the called peaks.

Data access links<br>*May remain private before publication.*	The datasets of ChIP-Seq (Project ID: PRJNA1201173, accession GSE285103) were deposited in the NCBI GEO database.
Files in database submission	CENH3-ChIP and input datasets:<br>ClRyeCENH3_EKDL220010420-1A_H3NGMDSX5_L2_1.fq.gz<br>ClRyeCENH3_EKDL220010420-1A_H3NGMDSX5_L2_2.fq.gz<br><br>ClRyeinput_EKDL220010419-1A_H3NM7DSX5_L1_1.fq.gz<br>ClRyeinput_EKDL220010419-1A_H3NM7DSX5_L1_2.fq.gz<br>ClRyeinput_EKDL220010419-1A_H5JCJDSX5_L4_1.fq.gz<br>ClRyeinput_EKDL220010419-1A_H5JCJDSX5_L4_2.fq.gz

CENH3_Input_1kb.bigwig

H3K4me2- and H3K9me2-ChIP and input datasets:
CLF-K4m2_L1_1.fq.gz
CLF-K4m2_L1_2.fq.gz
CLF-K9m2_L1_1.fq.gz
CLF-K9m2_L1_2.fq.gz
CLF-input_L1_1.fq.gz
CLF-input_L1_2.fq.gz

H3K9m2_input_1Kb.bigwig
H3K4m2_input_1Kb.bigwig

Genome browser session (e.g. UCSC): No longer applicable

Methodology

Replicates: All CENH3-, H3K9me2- and H3K4me2-ChIPseq experiments were performed for one biological replicate.

Sequencing depth: For all CENH3-, H3K9me2- and H3K4me2-ChIPseq experiments, at least 6 Gb of paired-end 150 bp illumina raw reads were generated.

Antibodies: Chamaelirium luteum-specific rabbit anti-ClCENH3 antibody, the commercially available rabbit anti-histone H3K4me2 (abcam, UK, cat. no. ab7766), and mouse anti-histone H3K9me2 (abcam, UK, cat. no. ab1220).

Peak calling parameters: The aim of the ChIPseq experiment was to determine the centromere-associated DNA sequences by CENH3-ChIP and the large-scale genome organization by H3K9me2- and H3K4me2-ChIP. The paired-end reads of ChIP- and input-seq were quality-filtered by Trimmmomatic (Galaxy Version 0.39) and the resulting reads were mapped to the Cha. luteum genome assembly using Bowtie2 (Galaxy Version 2.5.3) with default parameters. Peak calling (--broad -887400000) was performed by MACS (v3.0.1). The deeptools bamCompare (Galaxy Version 3.5.4) was used to generate ChIP-seq signal track as the average log2-ratio of ChIP over input read counts in genome-wide 1 kb windows. Visualization of chromosome regions with multiple tracks was plotted with pyGenomeTracks (Galaxy version 3.8).

Data quality: The CENH3-, H3K9me2-, and H3K4me2-ChIPseq patterns were consistent with our cytological observations, including the colocalization of CENH3-immuno and Chama-FISH signals, and the immunostaining patterns of H3K9me2 and H3K4me2.

Software: ChIPseq datasets were analyzed using ChIP-Seq Mapper Galaxy Version 1.1.1.4 (https://repeatexplorer-elixir.cerit-sc.cz/galaxy/), Bowtie2 Galaxy Version 2.5.3 (https://galaxy.ipk-gatersleben.de/), MACS Version 3.0.1 (https://github.com/macs3-project/MACS) and deeptools Galaxy Version 3.5.4. Genome browser tracks were produced using pyGenomeTracks Galaxy Version 3.8 (https://github.com/deeptools/pyGenomeTracks).

Flow Cytometry

Plots

Confirm that:

- [x] The axis labels state the marker and fluorochrome used (e.g. CD4-FITC).
- [x] The axis scales are clearly visible. Include numbers along axes only for bottom left plot of group (a 'group' is an analysis of identical markers).
- [ ] All plots are contour plots with outliers or pseudocolor plots.
- [ ] A numerical value for number of cells or percentage (with statistics) is provided.

Methodology

Sample preparation: Genome size measurement: Nuclei were isolated by manual chopping of young leaf tissue using a sharp razor blade in nuclei isolation buffer (CyStain PI Absolute P; Sysmex-Partec) and subsequent filtering through a 50 µm mesh (CellTrics, Sysmex-Partec).

Instrument: Genome size measurement: Sysmex CyFlow Space flow cytometer (Sysmex-Partec, Germany)

Software: Genome size measurement: FloMax Operating and Analysis Software for Flow Cytometry Particle Analysing Systems, Version 2.82

Cell population abundance: Genome size measurement: Based on the applied threshold setting (590LP) for the green laser (532 nm) the abundance of nuclei within the suspensions was above 70 %.

Gating strategy: Genome size measurement: Nuclei were separated from cellular debris by plotting the log-scale relative fluorescence intensity of the propidium iodide (PI) staining against the log-scale side scatter (SSC) signal using a green laser (532 nm) for excitation. The G1 peaks of Cha. luteum and the internal reference standard (Glycine max) were identified in the

corresponding histogram displaying the lin-scale PI relative fluorescence intensity of the nuclear fraction.

☒ Tick this box to confirm that a figure exemplifying the gating strategy is provided in the Supplementary Information.
